# Supplementary material for: Comprehensive single-cell sequencing reveals the stromal dynamics and tumor-specific characteristics in the microenvironment of nasopharyngeal carcinoma
Source: Nat Commun. 2021 Mar 9;12:1540. doi: 10.1038/s41467-021-21795-z (PMC7943808; doi:10.1038/s41467-021-21795-z)
Supplement: Supplementary file 1 — Supplementary Information [file 41467_2021_21795_MOESM1_ESM.pdf]

Supplementary Information for

Comprehensive single-cell sequencing reveals the stromal dynamics and tumor-specific characteristics in the microenvironment of nasopharyngeal carcinoma

Lanqi Gong[1, 2], Dora Lai-Wan Kwong[1, 2], Wei Dai[1, 2], Pingan Wu[3], Shanshan Li[1], Qian Yan[1, 2], Yu Zhang[2], Baifeng Zhang[2], Xiaona Fang[2], Li Liu[4, 5, 6], Min Luo[1], Beilei Liu[2], Larry Ka-Yue Chow[2], Qingyun Chen[7], Jinlin Huang[2], Victor Ho-Fun Lee[1, 2], Ka-On Lam[1, 2], Anthony Wing-Ip Lo[8], Zhiwei Chen[4, 5, 6], Yan Wang[9], Anne Wing-Mui Lee,[1, 2] Xin-Yuan Guan[1, 2, 7]

[1]Department of Clinical Oncology, The University of Hong Kong-Shenzhen Hospital, Shenzhen, China. [2]Department of Clinical Oncology, Li Ka Shing Faculty of Medicine, The University of Hong Kong, Hong Kong, China. [3]Department of Surgery, The University of Hong Kong-Shenzhen Hospital, Shenzhen, China. [4]Department of Microbiology, [5]The AIDS Institute, and [6]State Key Laboratory of Emerging Infectious Disease, Li Ka Shing Faculty of Medicine, The University of Hong Kong, Hong Kong, China. [7]State Key Laboratory of Oncology in Southern China, Sun Yat-sen University Cancer Center, Guangzhou, China. [8]Department of Pathology, Li Ka Shing Faculty of Medicine, The University of Hong Kong, Hong Kong, China. [9]Department of Pathology, The University of Hong Kong-Shenzhen Hospital, Shenzhen, China.

Correspondence: Xin-Yuan Guan, Department of Clinical Oncology, The University of Hong Kong-Shenzhen Hospital, 1 Haiyuan 1st Road, Futian District, Shenzhen, China 518053. Tel: 852-3917-9782; E-mail: xyguan@hku.hk

Table of Contents

Supplementary Figures 1-10

Supplementary Tables 1-3

Antibody List

a

Discovery cohort														
Disease Type	NPC											NLH		
Patient ID	1	2	3	4	5	6	7	8	9	10	14	11	12	13
Estimated Number of Cells	2,652	4,583	6,419	5,568	4,334	6,100	4,813	1,933	8,821	6,671	4,083	5,873	7,485	5,337
Mean Reads per Cell	182,645	101,428	75,836	108,071	122,944	71,949	92,561	132,696	53,390	67,921	156,846	76,830	63,222	90,004
Median Genes per Cell	1,331	1,398	727	1,185	1,144	704	581	643	1,760	1,424	1,300	1,543	1,602	1,670
Number of Reads	484,376,867	464,848,632	486,791,842	601,742,766	532,843,491	438,889,767	445,493,828	256,501,517	470,955,309	453,099,133	640,401,213	451,219,695	473,217,673	480,350,056
Valid Barcodes	92.30%	92.40%	94.30%	89.10%	87.60%	87.20%	87.60%	87.30%	91.90%	89.30%	85.60%	90.80%	91.90%	90.70%
Sequencing Saturation	95.50%	88.60%	94.60%	90.40%	90.10%	91.80%	93.40%	100.00%	77.30%	86.90%	92.00%	86.60%	81.20%	85.20%
Q30 Bases in Barcode	97.10%	97.00%	96.60%	96.60%	96.70%	96.40%	96.80%	96.80%	97.30%	97.40%	96.40%	97.40%	97.40%	97.40%
Q30 Bases in RNA Read	85.90%	88.10%	90.70%	94.60%	94.50%	89.20%	92.20%	83.70%	92.10%	92.00%	91.50%	92.10%	92.20%	92.00%
Q30 Bases in RNA Read 2	91.60%	91.90%	90.40%	89.80%	90.70%	94.10%	94.30%	94.00%	92.60%	91.90%	93.10%	92.00%	92.60%	92.40%
Q30 Bases in UMI	96.90%	96.70%	96.30%	95.80%	96.00%	96.30%	96.10%	96.80%	97.20%	97.20%	96.30%	97.30%	97.20%	97.30%
Reads Mapped to Genome	89.90%	89.50%	91.10%	85.60%	91.40%	89.50%	89.20%	75.90%	92.10%	91.80%	93.30%	91.60%	90.70%	92.60%
Reads Mapped Confidently to Genome	84.50%	80.20%	87.80%	74.90%	80.30%	81.70%	79.30%	69.30%	82.90%	81.00%	84.30%	81.70%	81.10%	84.20%
Reads Mapped Confidently to Intergenic Regions	8.80%	9.00%	4.80%	10.20%	11.20%	6.90%	4.70%	7.50%	6.90%	8.40%	9.80%	7.90%	6.90%	8.70%
Reads Mapped Confidently to Intronic Regions	5.00%	4.80%	6.50%	6.00%	7.00%	9.20%	9.30%	11.10%	5.90%	7.40%	7.50%	6.80%	5.70%	7.10%
Reads Mapped Confidently to Exonic Regions	73.50%	69.10%	79.20%	61.30%	64.10%	65.50%	65.30%	50.70%	72.60%	67.80%	66.90%	69.50%	70.90%	70.90%
Reads Mapped Confidently to Transcriptome	68.40%	64.50%	73.70%	56.50%	58.90%	53.80%	54.60%	41.50%	67.70%	62.00%	57.00%	64.40%	66.30%	65.40%
Reads Mapped Antisense to Gene	2.70%	2.30%	2.80%	2.40%	2.70%	7.80%	6.80%	6.00%	2.60%	3.30%	5.80%	2.70%	2.40%	3.00%
Fraction Reads in Cells	89.80%	97.30%	93.00%	95.70%	78.70%	73.80%	63.40%	72.80%	93.20%	89.50%	69.50%	89.80%	95.80%	81.20%
Total Genes Detected	20,126	21,156	20,156	21,369	21,292	20,395	19,298	17,767	21,649	20,364	20,138	21,609	21,866	20,613
Median UMI Counts per Cell	3,579	3,322	1,511	2,851	2,730	1,266	1,048	1,173	4,553	3,833	3,505	4,602	4,836	4,911

b

Validation cohort					
Disease Type	NPC		NLH		
Patient ID	9	10	11	12	13
Estimated Number of Cells	10,092	7,307	8,150	8,653	7,893
Mean Reads per Cell	63,930	93,319	92,317	81,248	88,885
Median Genes per Cell	1,701	1,565	1,713	1,634	1,696
Number of Reads	645,180,272	681,882,183	752,379,807	703,036,290	701,567,034
Valid Barcodes	85.00%	82.10%	84.10%	84.70%	83.30%
Sequencing Saturation	75.70%	86.10%	85.20%	81.80%	82.20%
Q30 Bases in Barcode	97.10%	97.10%	97.10%	97.10%	97.10%
Q30 Bases in RNA Read	91.80%	91.20%	91.50%	92.00%	91.40%
Q30 Bases in RNA Read 2	93.10%	95.40%	95.80%	96.10%	95.70%
Q30 Bases in UMI	96.70%	96.70%	96.80%	96.60%	96.70%
Reads Mapped to Genome	92.50%	92.80%	93.30%	91.80%	93.40%
Reads Mapped Confidently to Genome	84.00%	84.20%	86.20%	83.20%	85.60%
Reads Mapped Confidently to Intergenic Regions	7.60%	8.80%	8.00%	7.90%	8.60%
Reads Mapped Confidently to Intronic Regions	18.70%	22.60%	22.60%	18.10%	22.10%
Reads Mapped Confidently to Exonic Regions	57.70%	52.90%	55.60%	57.10%	54.90%
Reads Mapped Confidently to Transcriptome	44.80%	39.40%	42.30%	44.20%	41.40%
Reads Mapped Antisense to Gene	9.20%	9.70%	9.30%	9.30%	9.60%
Fraction Reads in Cells	89.50%	88.20%	92.00%	94.70%	86.10%
Total Genes Detected	23,460	22,334	23,405	23,129	22,506
Median UMI Counts per Cell	3,760	3,556	4,314	4,158	4,334

c

B cell					
Discovery			Validation		
Cluster	Count	Frequency	Cluster	Count	Frequency
Unactivated	4501	0.163637	Unactivated	4480	0.23069
Naïve	5975	0.217225	Naïve	5708	0.293924
Innate-like	4839	0.175925	Innate-like	2409	0.124047
IFN-induced	2918	0.106086	*IFN*-induced	1705	0.087796
Plasma	2900	0.105432	Plasma	2044	0.105252
Switched memory	1708	0.062096	Switched memory	1006	0.051802
Double-negative	1658	0.060278	Double-negative	1351	0.069567
IFN-induced naïve	1251	0.045481	*IFN*-induced naïve	439	0.022606
Germinal-center	860	0.031266	Germinal-center	255	0.013131
Proliferating	632	0.022977	Proliferating	10	0.000515
EBV-induced	264	0.009598	EBV-induced	13	0.000669

e

T cell					
Discovery			Validation		
Cluster	Count	Frequency	Cluster	Count	Frequency
CD4 naïve	5875	0.179906	CD4 naïve	5022	0.28887
CD8 naïve	728	0.022293	CD8 naïve	649	0.037331
CD8 activated	3434	0.105157	CD8 activated	1337	0.076905
CD4 T helper	3065	0.093857	CD4 T helper	1844	0.106068
CD4 suppressive Treg	2807	0.085957	CD4 suppressive Treg	1317	0.075755
CD8 HAVCR2 exhaustec	2739	0.083874	CD8 *HAVCR2* exhausted	1654	0.095139
CD4 resting Treg	2645	0.080996	CD4 resting Treg	456	0.02623
CD8 Trm	2471	0.075668	CD8 Trm	1044	0.060052
CD8 TOX exhausted	1941	0.059438	CD8 *TOX* exhausted	1399	0.080472
CD4 central memory	1884	0.057692	CD4 central memory	669	0.038481
CD8 Tem	1622	0.049669	CD8 Tem	837	0.048145
Proliferating	1504	0.046056	Proliferating	651	0.037446
CD8 exhausted Trm	1228	0.037604	CD8 exhausted Trm	369	0.021225
CD4 IFN-stimulated	713	0.021834	CD4 *IFN*-stimulated	137	0.00788

d

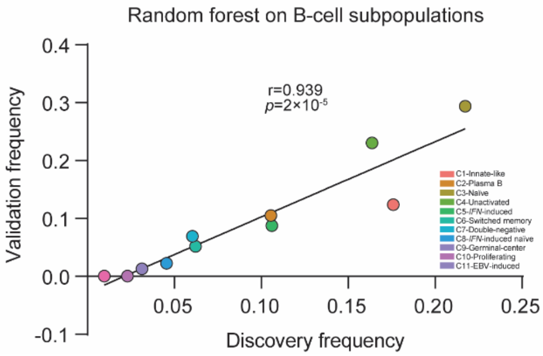

f

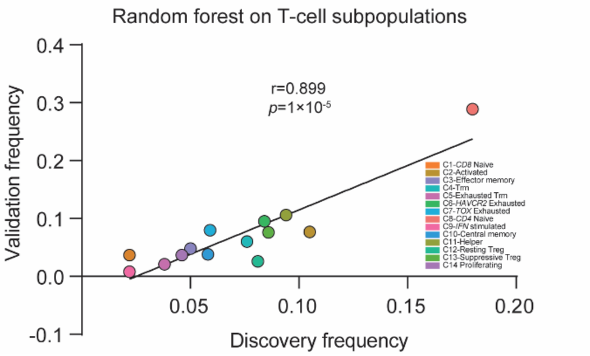

Supplementary Fig. 1 Quality control reports for each sequenced sample and independent validation via the random forest. (a) The summary of single-cell sequencing quality control report in the discovery cohort. (b) The summary of single-cell

sequencing quality control report in the validation cohort. (c) The random forest analysis performed on B cells (d) The Pearson correlation between the frequency of B cell subpopulations in the discovery cohort and validation cohort, color-coded by the patient ID. (e) The random forest analysis performed on T cells (f) The Pearson's correlation between the frequency of T cell subpopulations in the discovery cohort and validation cohort, color-coded by the patient ID. Source data are provided as a Source Data file.

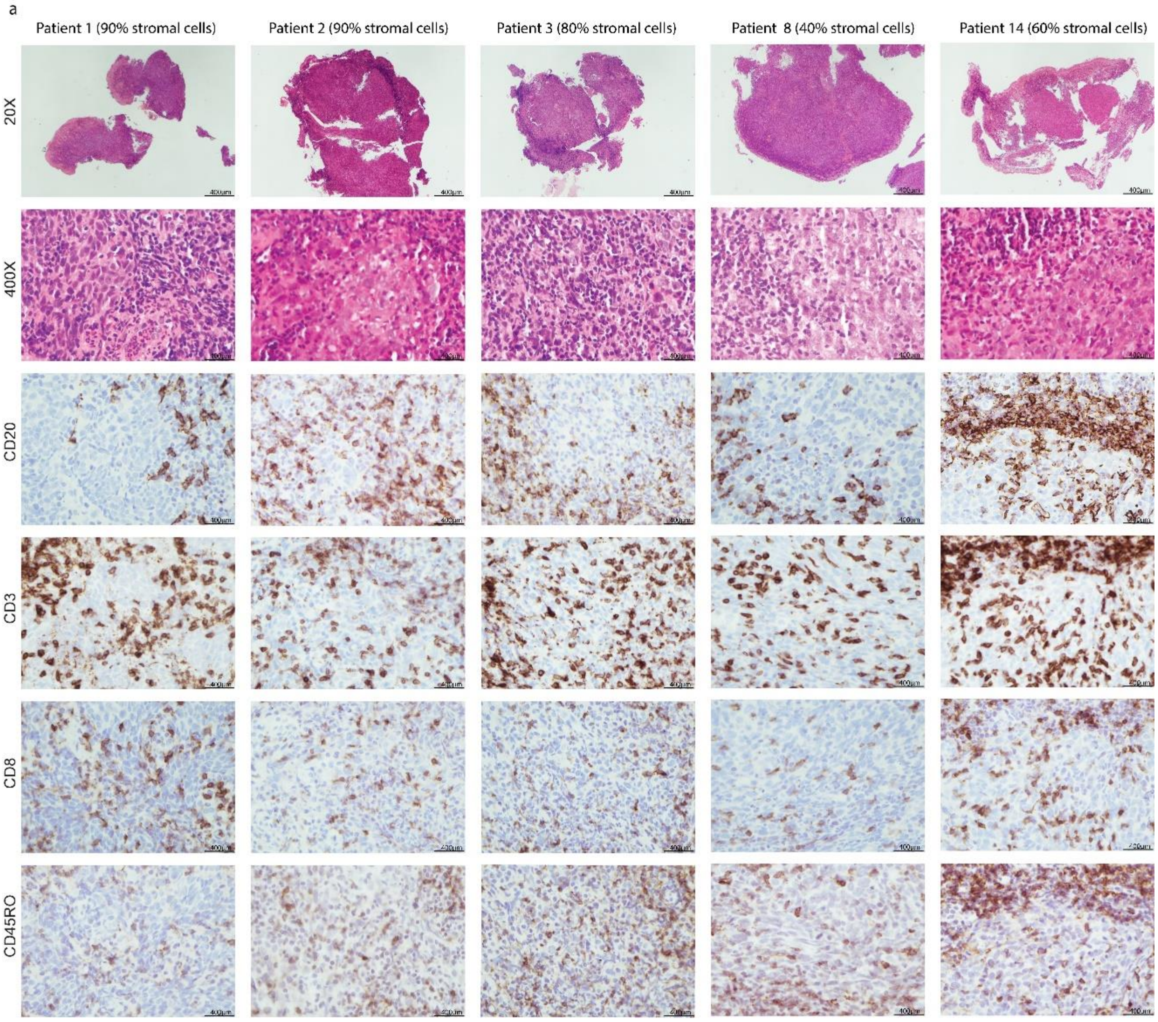

Supplementary Fig. 2 Degree of stromal infiltration in NPC patients determined by pathological examination. (a) The representative H&E (20X and 400X) and IHC staining (400X) pictures of NPC patients, showing the degree of stromal infiltration, evaluated by a group of professional pathologists. Three independent experiments were performed and generated similar results. Scale bar = 400 µm.

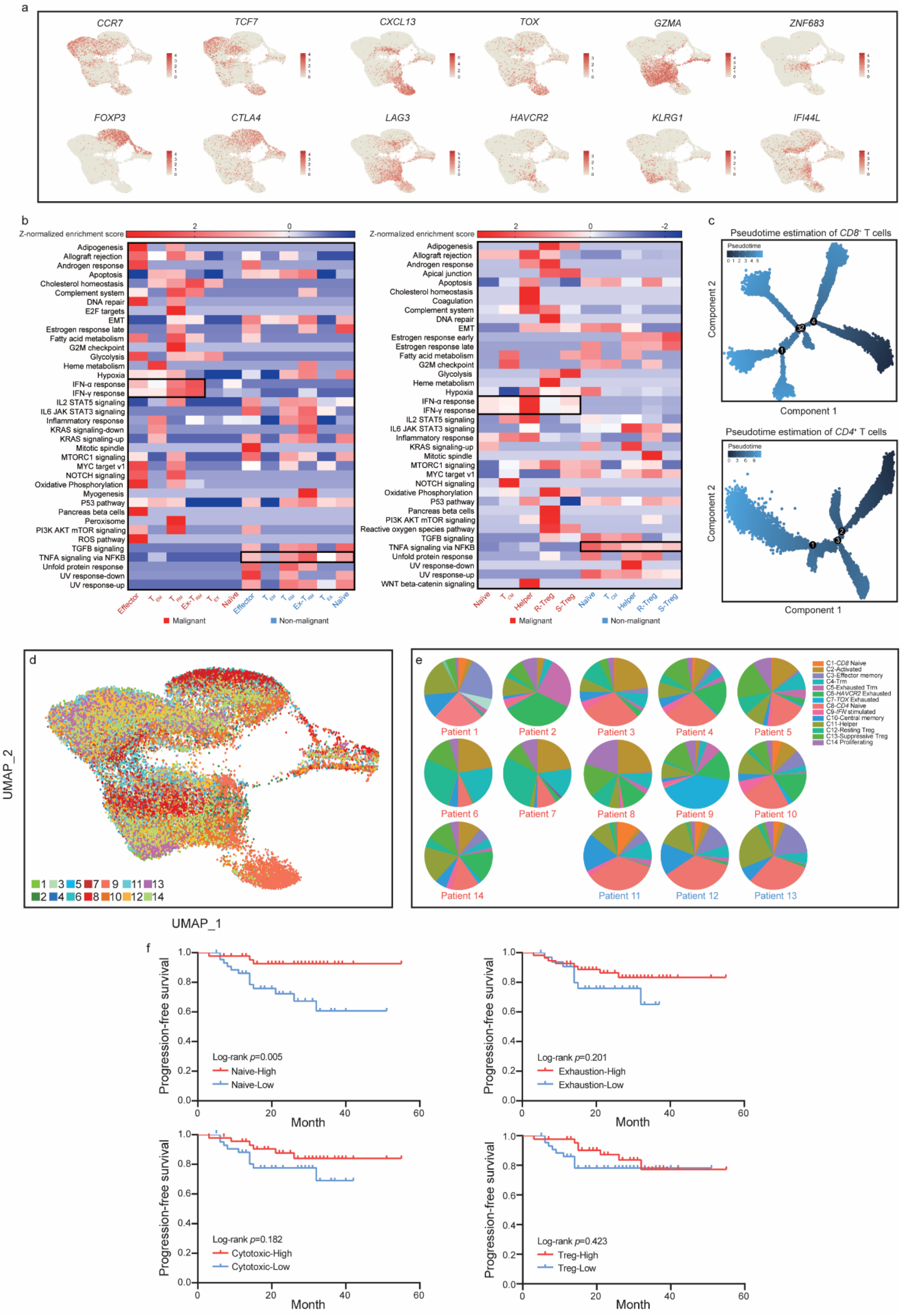

Supplementary Fig. 3 T-cell associated signatures and correlations to progression-free survival. (a) The expression of marker genes for T cell subpopulations defined in fig. 2a. (b) The GSEA hallmark pathways enriched in *CD8*$^+$ and *CD4*$^+$ T cell subpopulations derived from the malignant and non-malignant microenvironment. (c) The pseudotime estimation in the developmental trajectory of *CD8*$^+$ and *CD4*$^+$ T cells. (d) The patient distribution plot within the T cell subpopulations, color-coded by the patient ID. (e) The inter-patient distribution of T cell subpopulations, shown by the percentage of total T cells. (f) The progression-free survival for 88 NPC patients, stratified for the naïve, cytotoxic, Treg and exhaustion scores (binary: high score vs. low score), calculated by the corresponding linear models, respectively. The *p*-values were evaluated by the two-sided log-rank test. Source data are provided as a Source Data file.

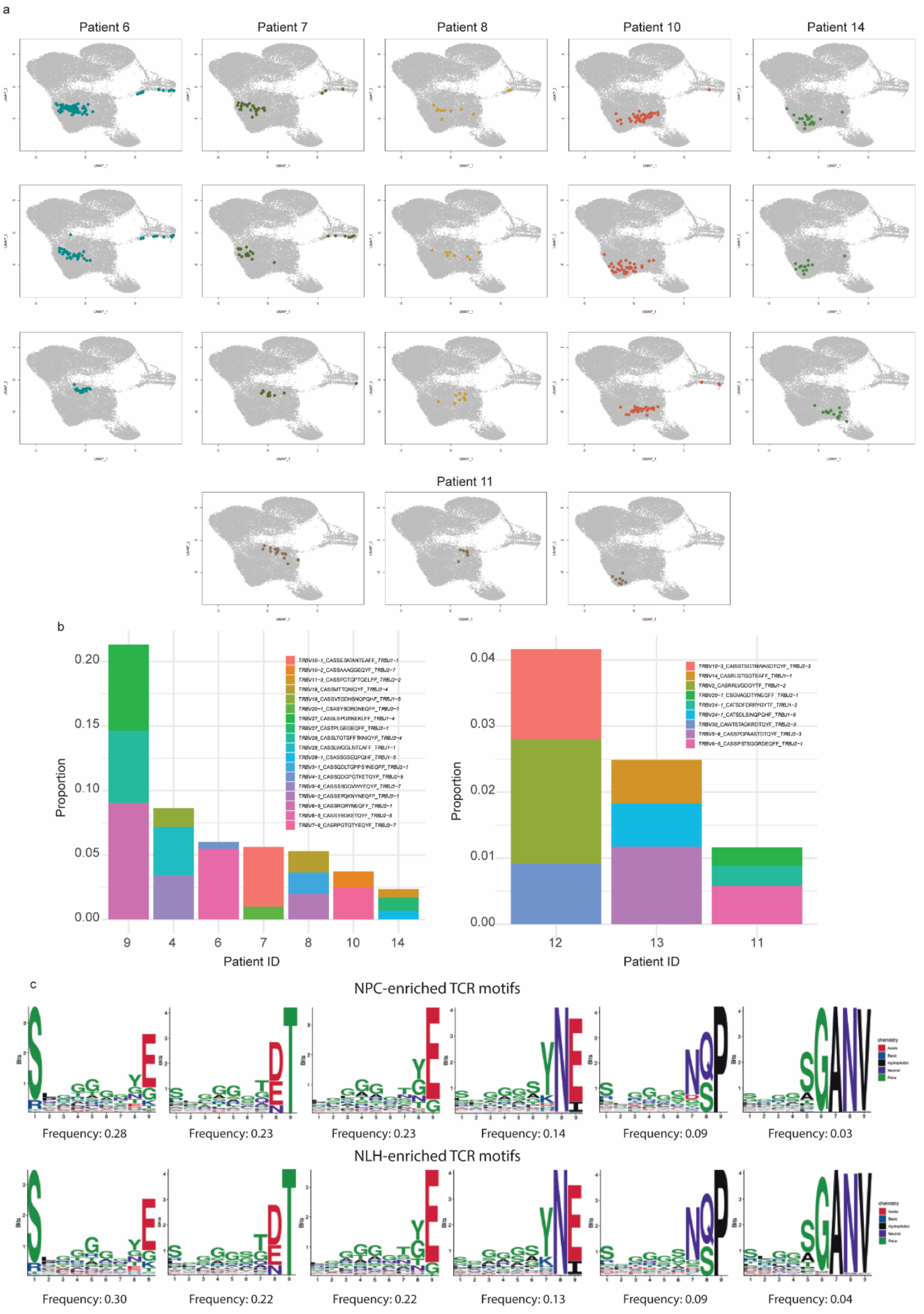

a
Patient 6
Patient 7
Patient 8
Patient 10
Patient 14
Patient 11
b
0.20
0.15
0.10
0.05
0.00
Proportion
TRBV10-1_CASSESATANTEAFF_TRBJ1-1
TRBV10-2_CASSAAAGGEQYF_TRBJ2-7
TRBV11-3_CASSPGTGPTGELFF_TRBJ2-2
TRBV19_CASSMTTQNIQYF_TRBJ2-4
TRBV19_CASSVTGDHSNQPQHF_TRBJ1-5
TRBV20-1_CSASYSGRGNEQFF_TRBJ2-1
TRBV27_CASSLSPGRNEKLFF_TRBJ1-4
TRBV27_CASTPLGEGEQFF_TRBJ2-1
TRBV28_CASSLTGTSFFTKNIQYF_TRBJ2-4
TRBV28_CASSLWQGLNTEAFF_TRBJ1-1
TRBV29-1_CSASSGSEQPQHF_TRBJ1-5
TRBV3-1_CASSQDLTGPPSYNEQFF_TRBJ2-1
TRBV4-3_CASSQDGPGTKETQYF_TRBJ2-5
TRBV5-6_CASSSSGGVWYEQYF_TRBJ2-7
TRBV6-2_CASSEPQKNYNEQFF_TRBJ2-1
TRBV6-5_CASSRGRYNEQFF_TRBJ2-1
TRBV6-5_CASSYEGKETQYF_TRBJ2-5
TRBV7-9_CASRPGTGTYEQYF_TRBJ2-7
9 4 6 7 8 10 14
Patient ID
0.04
0.03
0.02
0.01
0.00
Proportion
TRBV10-3_CAISSTSGTNWASDTQYF_TRBJ2-3
TRBV14_CASRLGTGGTEAFF_TRBJ1-1
TRBV2_CASRRLVGDGYTF_TRBJ1-2
TRBV20-1_CSGVAGDTYNEQFF_TRBJ2-1
TRBV24-1_CATSDFDRRYGYTF_TRBJ1-2
TRBV24-1_CATSDLSINQPQHF_TRBJ1-5
TRBV30_CAWTSTAGKRDTQYF_TRBJ2-3
TRBV5-6_CASSPGPAASTDTQYF_TRBJ2-3
TRBV6-5_CASSPSTSGGRDEQFF_TRBJ2-1
12 13 11
Patient ID
c
NPC-enriched TCR motifs
Frequency: 0.28
Frequency: 0.23
Frequency: 0.23
Frequency: 0.14
Frequency: 0.09
Frequency: 0.03
NLH-enriched TCR motifs
Frequency: 0.30
Frequency: 0.22
Frequency: 0.22
Frequency: 0.13
Frequency: 0.09
Frequency: 0.04

Supplementary Fig. 4 Identification of T cell clonality and T cell receptor motifs. (a) UMAP plot of the top 3 largest clones in patients with their corresponding clonotypes. (b) The proportion of the top 3 frequent clonotypes in patients, color-coded by associated clonotypes. (c) The enriched amino acid motifs on the TCR-β chains of NPC and NLH-derived T cells, ordered by the frequency, color-coded by the biochemical property, scaled by enrichment score.

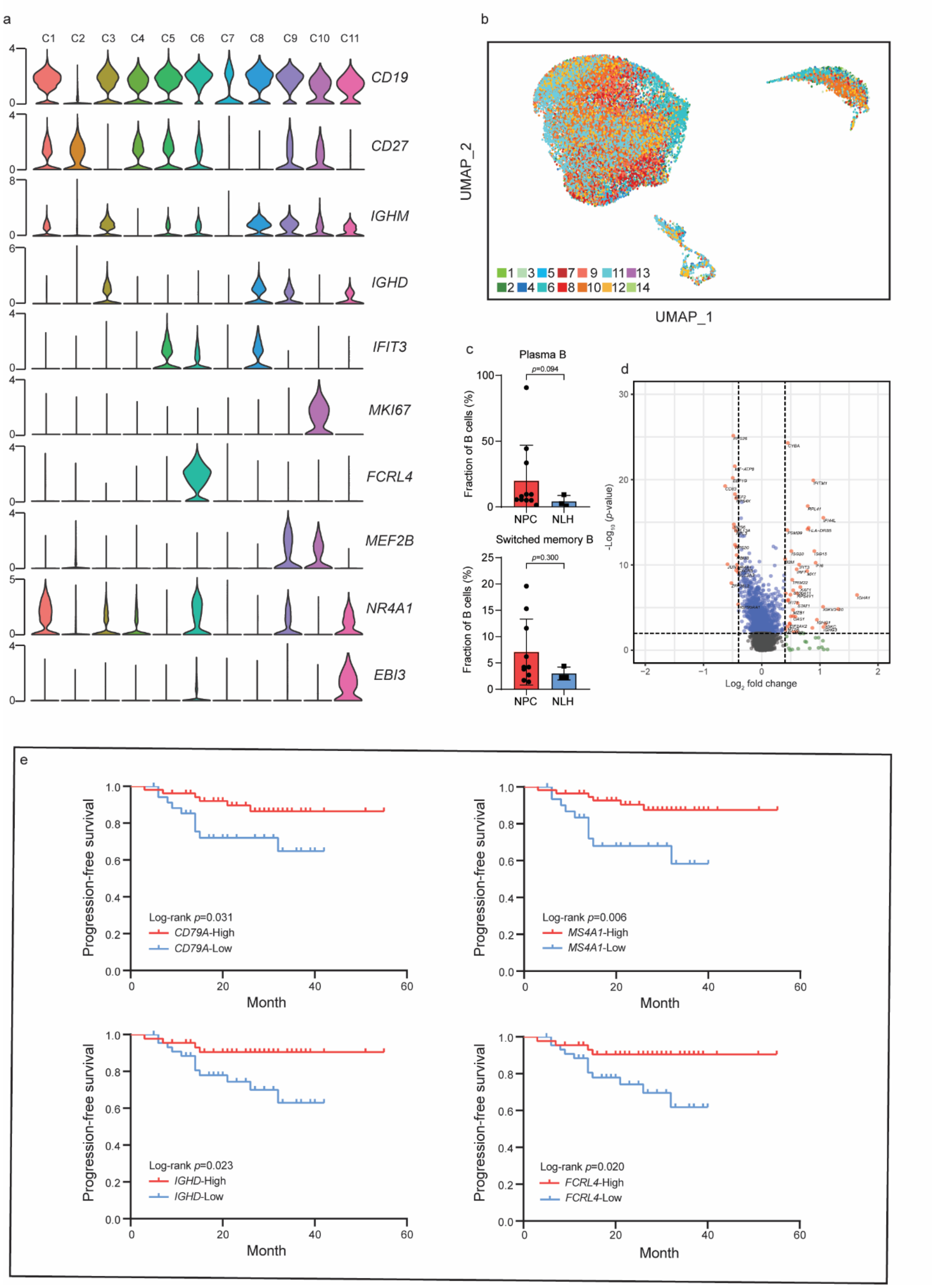

Supplementary Fig. 5 B-cell associated signatures and correlations to progression-free survival. (a) Violin plots showing the average expression of marker genes and

functional signatures in the B cell subpopulations defined in fig. 5a. (b) The patient distribution plot within the B cell subpopulations, colored by the patient ID. (c) The relative abundance of plasma B cells and switched memory B cells in the NPC (n=11 biologically independent samples) and NLH (n=3 biologically independent samples) microenvironment. Each dot represents one patient. *p*-values were evaluated using two-sided Student's t-test. Data are presented as mean values ±SD. (d) The differentially expressed genes ($\log_2$ fold change≥0.4, *p*-values≤5×10^{-2}) in NPC-derived and NLH-derived B cells identified by the MAST analysis. (e) The progression-free survival for 88 NPC patients, stratified for the normalized average expression (binary: high expression vs. low expression) of a selected set of B-cell signature genes. *p*-values were evaluated by the two-sided log-rank test. Source data are provided as a Source Data file.

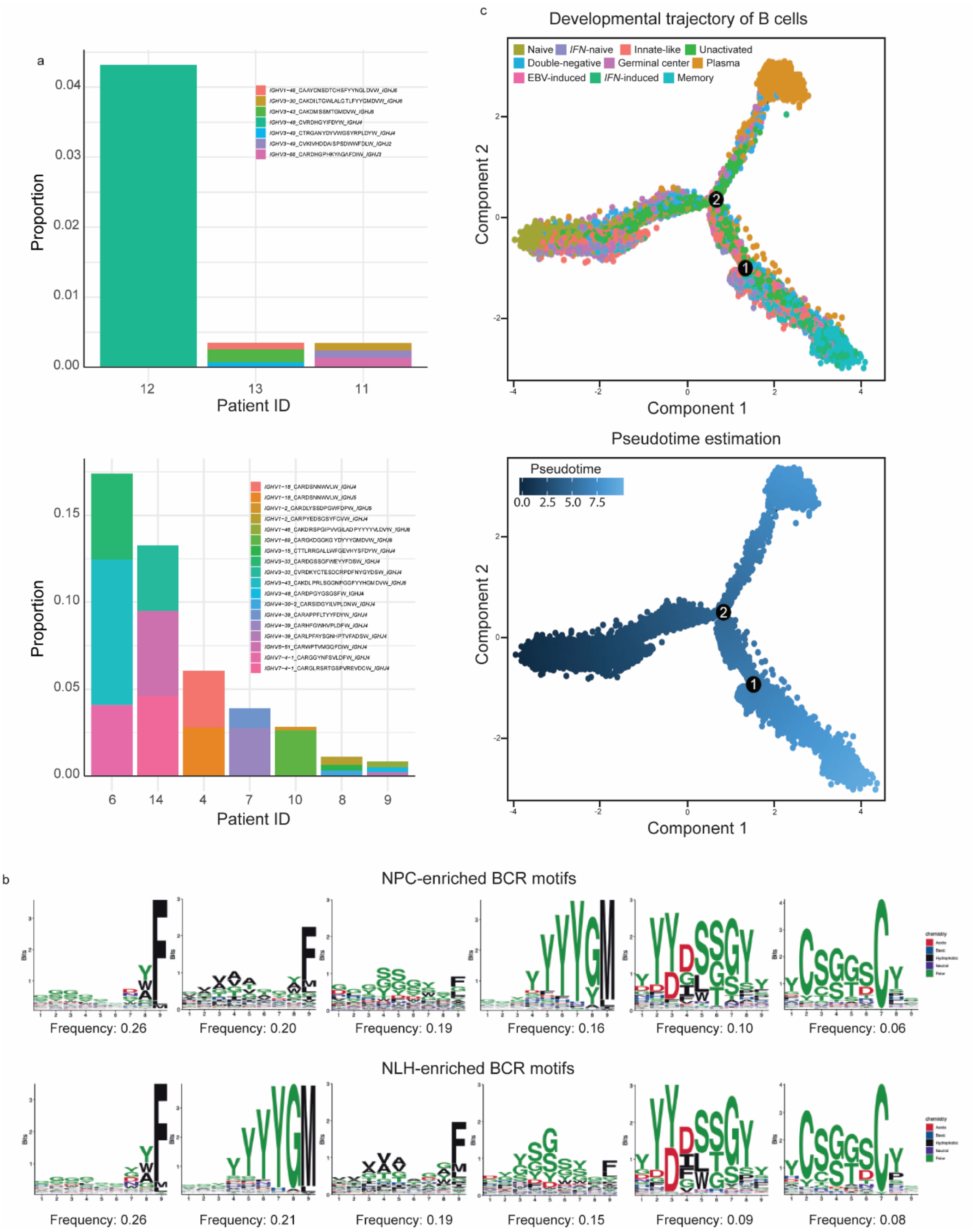

Supplementary Fig. 6 Identification of B cell clonality and B cell receptor motifs. (a) The proportion of the top 3 frequent clonotypes in patients, colored by associated clonotypes. (b) The enriched amino acid motifs on the BCR-β chains of NPC and NLH-derived B cells, ordered by the frequency, colored by the biochemical property, scaled by enrichment score. (c) The pseudotime developmental trajectory of B cells based on the expression of the top 50 marker genes in each subpopulation.

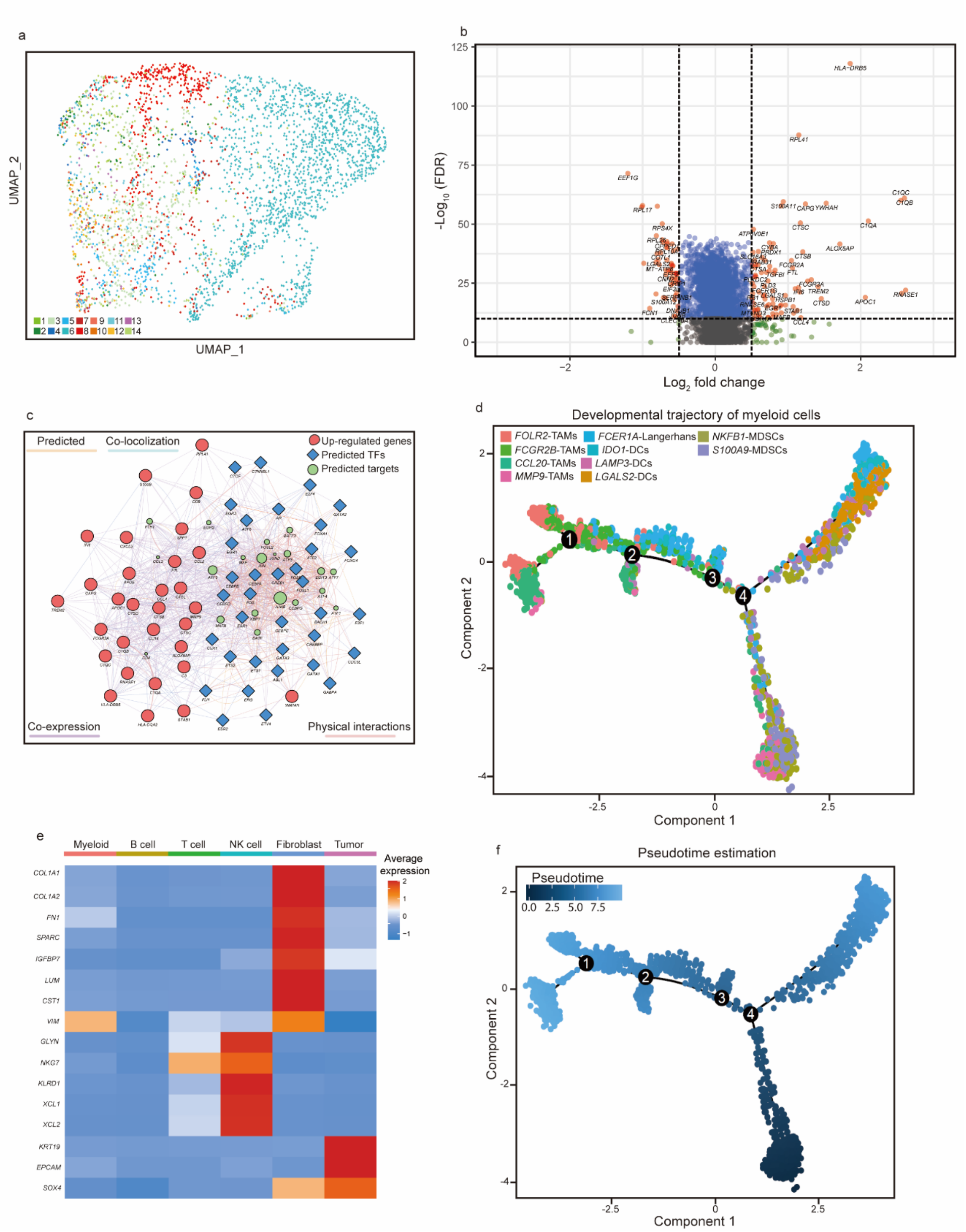

Supplementary Fig. 7 Genetic signatures in myeloid cells, NK cells, fibroblasts and tumor cells. (a) The patient distribution plot within the myeloid subpopulations, color-coded by the patient ID. (b) The differentially expressed genes ($\log_2$ fold change ≥ 0.5, FDR $\leq 1\times10^{-20}$) in NPC-derived and NLH-derived myeloid cells identified by the MAST analysis. (c) The gene regulatory network constructed by Cytoscape, color-coded by the associated

gene type (red: top 30 up-regulated genes from NPC-derived myeloid cells, blue: the upstream transcription factors predicted by RegNetwork, green: the targeted genes predicted by GeneMANIA). (d and e) The pseudotime developmental trajectory of myeloid cells based on the expression of the top 30 marker genes in each subpopulation. (f) Heatmap showing the differentially expressed genes in fibroblasts, NK cells and tumor cells.

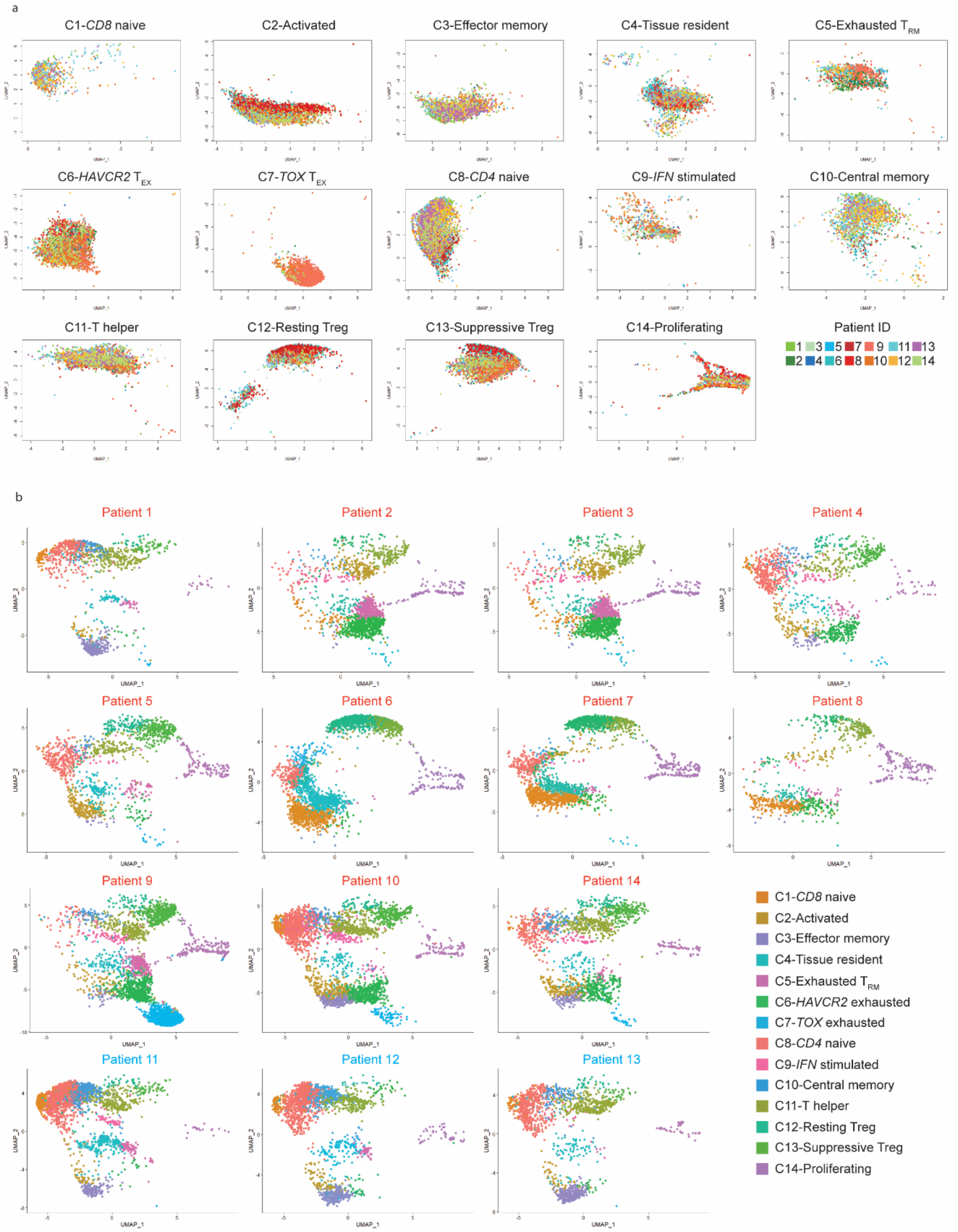

Supplementary Fig. 8 Patient and cell-type distribution within T cells. (a) UMAP plot showing the patient distribution in each T cell subtype, color-coded by the patient ID. (b) UMAP plot showing the T cell distribution in each patient, color-coded by the cell subtype.

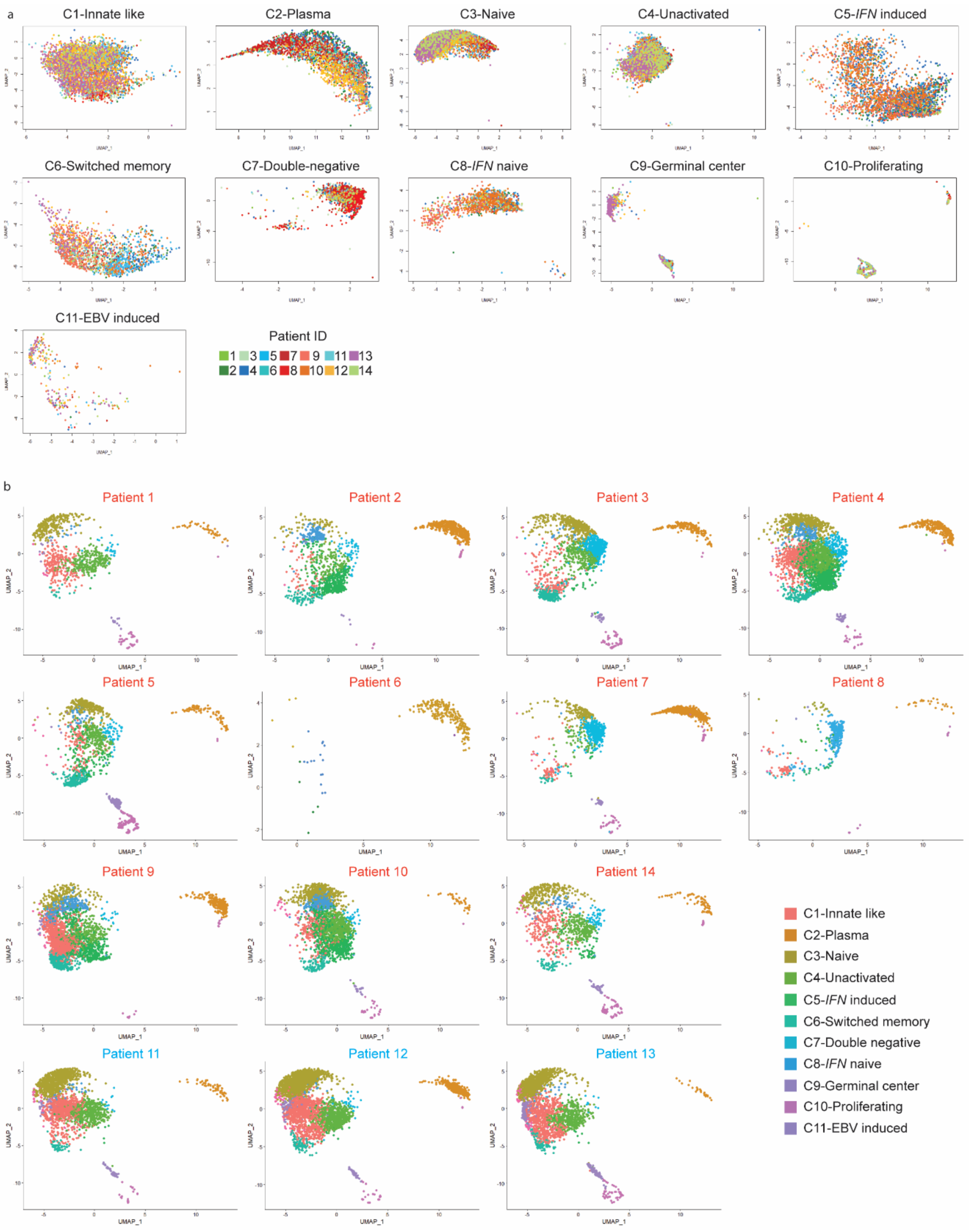

Supplementary Fig. 9 Patient and cell-type distribution within B cells. (a) UMAP plot showing the patient distribution in each B cell subtype, color-coded by the patient ID. (b) UMAP plot showing the B cell distribution in each patient, color-coded by the cell subtype.

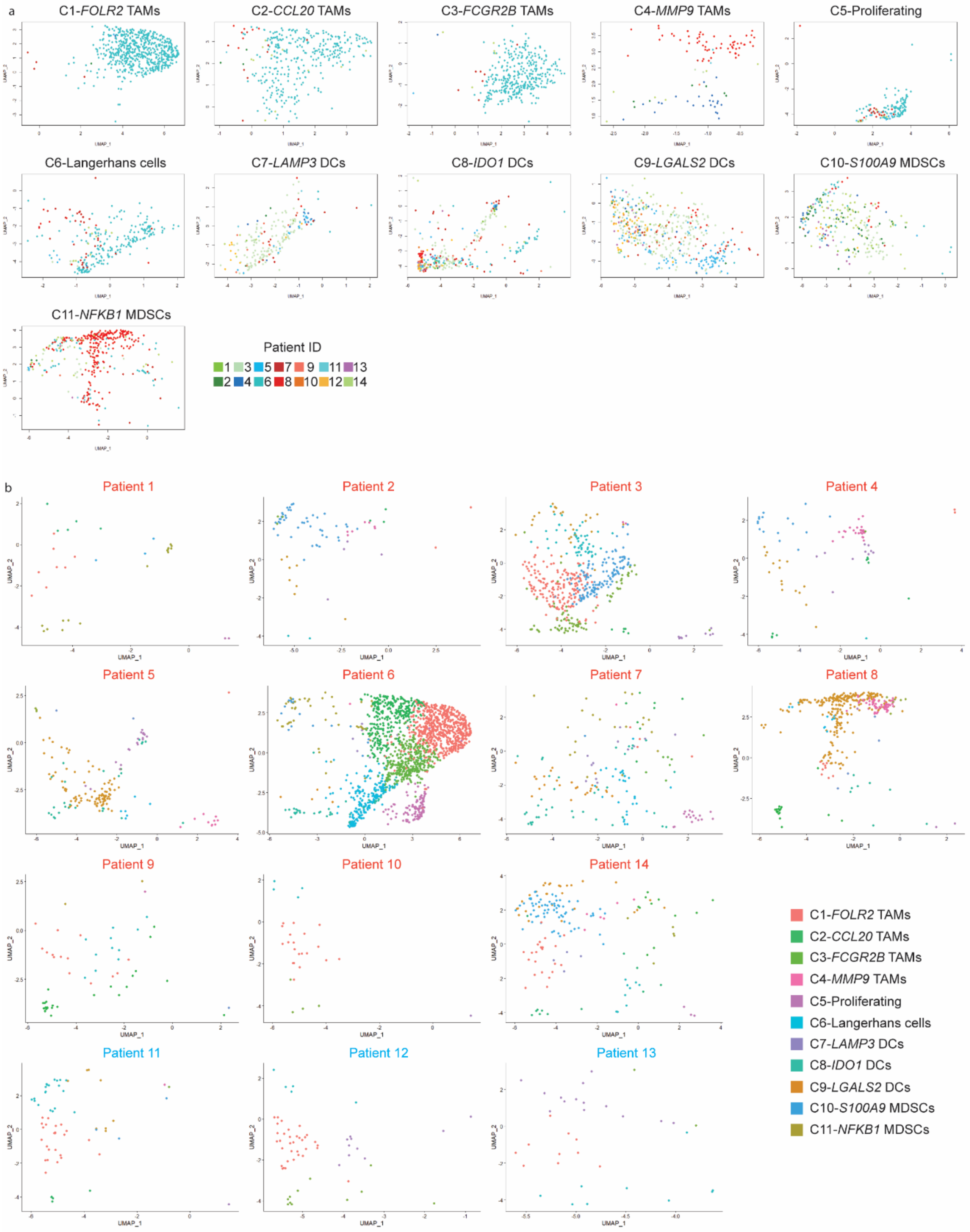

Supplementary Fig. 10 Patient and cell-type distribution within myeloid cells. (a) UMAP plot showing the patient distribution in each myeloid cell subtype, color-coded by the patient ID. (b) UMAP plot showing the myeloid cell distribution in each patient, color-coded by the cell subtype.

Patient ID	EBV status	Stage	Differentiation type
1	Negative	III	Undifferentiated
2	Positive	III	Undifferentiated
3	Positive	III	Undifferentiated
4	Positive	IV	Undifferentiated
5	Negative	I	Undifferentiated
6	Positive	IVB	Differentiated
7	Negative	III	Undifferentiated
8	N/A	IVA	Undifferentiated
9	Positive	III	Undifferentiated
10	Positive	III	Undifferentiated
11	Normal	Normal	Normal
12	Normal	Normal	Normal
13	Normal	Normal	Normal
14	Negative	III	Undifferentiated

Supplementary Table 1 The clinical information of 14 patients enrolled in the present study.

Patient	EBV reads	Stage	Grade	Event	Status	Time to event (m)
GZNPC_747	8815	NA	undifferentiated, round	Last follow-up	0	30
GZNPC_748	3829	IV	NA	Last follow-up	0	21
GZNPC_749	2583	III	mixed (round & spindle)	Disease progression	1	21
GZNPC_750	4719	NA	mixed (round & spindle)	Last follow-up	0	23
GZNPC_751	1703	II	differentiated	Last follow-up	0	29
GZNPC_753	3099	IV	mixed (round & spindle)	Last follow-up	0	27
GZNPC_754	6288	NA	differentiated	Last follow-up	0	39
GZNPC_756	2675	III	undifferentiated, round	Disease progression	1	6
GZNPC_757	20678	IV	differentiated	Last follow-up	0	40
GZNPC_758	5661	NA	mixed (round & spindle)	Last follow-up	0	38
GZNPC_760	3903	III	mixed (round & spindle)	Last follow-up	0	35
GZNPC_761	1143	NA	differentiated	Last follow-up	0	31
GZNPC_762	8294	IV	undifferentiated, spindle	Last follow-up	0	22
GZNPC_763	8185	III	undifferentiated, round	Disease progression	1	6
GZNPC_764	4526	III	undifferentiated, round	Last follow-up	0	27
GZNPC_765	1662	NA	undifferentiated, round	Last follow-up	0	25

GZNPC_766	19424	III	mixed (round & spindle)	Last follow-up	0	36
GZNPC_767	4848	III	mixed (round & spindle)	Last follow-up	0	29
GZNPC_768	2349	III	mixed (round & spindle)	Disease progression	1	8
GZNPC_769	3231	NA	differentiated	Last follow-up	0	36
GZNPC_771	17135	III	undifferentiated, round	Last follow-up	0	9
GZNPC_772	712	III	undifferentiated, spindle	Last follow-up	0	42
GZNPC_773	3525	III	NA	Last follow-up	0	15
GZNPC_774	2479	III	mixed (round & spindle)	Last follow-up	0	29
GZNPC_775	17316	NA	undifferentiated, round	Last follow-up	0	18
GZNPC_777	5290	IV	undifferentiated, round	Last follow-up	0	34
GZNPC_778	2050	IV	mixed (round & spindle)	Last follow-up	0	13
GZNPC_779	2600	NA	mixed (round & spindle)	Last follow-up	0	12
GZNPC_780	2345	III	mixed (round & spindle)	Last follow-up	0	37
GZNPC_781	8757	III	undifferentiated, round	Last follow-up	0	39
GZNPC_782	2322	IV	mixed (round & spindle)	Last follow-up	0	23
GZNPC_783	2329	IV	undifferentiated, round	Disease progression	1	11
GZNPC_784	3601	IV	NA	Disease progression	1	14
GZNPC_785	93	III	mixed (round & spindle)	Last follow-up	0	20
GZNPC_786	20875	NA	differentiated	Last follow-up	0	33
GZNPC_787	1720	III	mixed (round & spindle)	Last follow-up	0	51
GZNPC_788	2827	NA	undifferentiated, round	Disease progression	1	15
GZNPC_789	5719	III	mixed (round & spindle)	Last follow-up	0	32
GZNPC_790	2955	III	mixed (round & spindle)	Last follow-up	0	21
GZNPC_791	3939	III	undifferentiated, spindle	Disease progression	1	9
GZNPC_792	441	II	NA	Last follow-up	0	27
GZNPC_794	10076	NA	mixed (round & spindle)	Last follow-up	0	37
GZNPC_795	5176	NA	undifferentiated, spindle	Last follow-up	0	16
GZNPC_796	4986	IV	undifferentiated, spindle	Disease progression	1	32
GZNPC_797	4664	III	differentiated	Last follow-up	0	18
GZNPC_798	3985	IV	mixed (round & spindle)	Last follow-up	0	13
GZNPC_799	4148	IV	undifferentiated, round	Last follow-up	0	31
GZNPC_800	4607	III	mixed (round & spindle)	Last follow-up	0	29
GZNPC_802	3323	IV	NA	Last follow-up	0	37
GZNPC_803	17771	III	undifferentiated, spindle	Last follow-up	0	40
GZNPC_804	36680	NA	undifferentiated, round	Last follow-up	0	23
GZNPC_805	2180	III	mixed (round & spindle)	Last follow-up	0	20
GZNPC_806	5499	I	mixed (round & spindle)	Last follow-up	0	20
GZNPC_809	4779	III	undifferentiated, spindle	Last follow-up	0	17
GZNPC_810	2784	III	undifferentiated, round	Last follow-up	0	12
GZNPC_811	8904	III	mixed (round & spindle)	Last follow-up	0	36
GZNPC_812	544	NA	mixed (round & spindle)	Last follow-up	0	27

GZNPC_817	1930	I	NA	Last follow-up	0	29
GZNPC_818	3244	NA	undifferentiated, round	Last follow-up	0	27
GZNPC_819	7618	IV	differentiated	Disease progression	1	14
GZNPC_821	4725	III	NA	Last follow-up	0	55
GZNPC_822	1506	III	undifferentiated, round	Last follow-up	0	38
GZNPC_823	2898	III	mixed (round & spindle)	Last follow-up	0	33
GZNPC_824	479	III	NA	Disease progression	1	3
GZNPC_825	5582	I	undifferentiated, round	Last follow-up	0	31
GZNPC_826	2671	NA	undifferentiated, round	Last follow-up	0	7
GZNPC_829	3903	IV	mixed (round & spindle)	Last follow-up	0	14
GZNPC_830	5026	III	mixed (round & spindle)	Last follow-up	0	42
GZNPC_832	288	I	undifferentiated, round	Last follow-up	0	26
GZNPC_835	293	III	mixed (round & spindle)	Last follow-up	0	5
GZNPC_836	5739	NA	undifferentiated, round	Last follow-up	0	13
GZNPC_837	13799	NA	undifferentiated, round	Last follow-up	0	37
GZNPC_839	2725	NA	undifferentiated, round	Disease progression	1	15
GZNPC_840	6979	IV	mixed (round & spindle)	Disease progression	1	14
GZNPC_841	12539	IV	differentiated	Disease progression	1	26
GZNPC_842	328	IV	NA	Last follow-up	0	23
GZNPC_843	1967	III	undifferentiated, round	Last follow-up	0	22
GZNPC_844	2427	III	undifferentiated, round	Last follow-up	0	30
GZNPC_845	3648	IV	undifferentiated, round	Disease progression	1	14
GZNPC_848	5199	IV	mixed (round & spindle)	Last follow-up	0	27
GZNPC_849	2927	III	undifferentiated, spindle	Last follow-up	0	21
GZNPC_850	4903	IV	differentiated	Last follow-up	0	18
GZNPC_852	2450	IV	undifferentiated, round	Last follow-up	0	17
GZNPC_853	14604	III	undifferentiated, round	Last follow-up	0	19
GZNPC_855	2678	III	undifferentiated, round	Last follow-up	0	25
GZNPC_856	4119	III	mixed (round & spindle)	Last follow-up	0	28
GZNPC_858	216	I	NA	Last follow-up	0	12
GZNPC_859	2566	III	mixed (round & spindle)	Disease progression	1	7

Supplementary Table 2 The clinical information of 88 patients enrolled in GSE102349.

Patient	Stage
701T	III
702T	III
704T	III
706T	III
707T	III
708T	III
710T	III
711T	III
712T	III
713T	III
714T	III
716T	III
718T	III
719T	III
720T	III
722T	III
723T	III
724T	III
725T	II
728T	III
729T	III
730T	III
731T	II
732T	III
733T	II
735T	III
736T	II
737T	III
740T	II
741T	III
743T	II
744T	III
746T	III
747T	III
748T	III
749T	III
751T	III
752T	III
754T	III
755T	III

756T	III
757T	III
703N	NA
721N	NA
727N	NA

Supplementary Table 3 The clinical information of 45 patients enrolled in GSE68799.

Antibody List

Antibody	Company	Catalog No.	Dilution
IHC staining			
anti-CD20	Dako	M0755	1:2400
anti-CD3	Leica	CD3-565-L-CE	1:50
anti-CD8	Dako	M7103	1:160
anti-CD45RO	Dako	M0742	1:500
IF staining (primary antibody)			
anti-PD1	Abcam	ab52587	1:50
anti-CD8	Abcam	ab4055	1:200
anti-FOXP3	Abcam	ab450	1:500
anti-CD3	AbD Serotec	MCA 1477	1:100
IF staining (secondary antibody)			
Goat anti-mouse IgG, Alexa 488	Invitrogen	A-10680	1:1000
Goat anti-rabbit, Alexa 568	Invitrogen	A-11011	1:1000
Goat anti-rat IgG, Alexa 488	Invitrogen	A-11006	1:1000
